# High‐Performance Virus Removal Filter Paper for Drinking Water Purification

**DOI:** 10.1002/gch2.201800031

**Published:** 2018-07-11

**Authors:** Olof Gustafsson, Levon Manukyan, Albert Mihranyan

**Affiliations:** ^1^ Nanotechnology and Functional Materials Department of Engineering Sciences Uppsala University Box 534 ,751 21 Uppsala Sweden

**Keywords:** *Cladophora* sp. algae cellulose, drinking water, mille‐feuille filter, nanocellulose, water purification

## Abstract

Access to drinking water is one of the greatest global challenges today. In this study, the virus removal properties of mille‐feuille nanocellulose‐based filter papers of varying thicknesses from simulated waste water (SWW) matrix are evaluated for drinking water purification applications. Filtrations of standard SWW dispersions at various total suspended solid (TSS) content are performed, including spiking tests with 30 nm surrogate latex particles and 28 nm ΦX174 bacteriophages. Filter papers of thicknesses 9 and 29 µm are used, and the filtrations are performed at two different operational pressures, i.e., 1 and 3 bar. The presented data using SWW matrix show, for the first time, that a filter paper made from 100% nanocellulose has the capacity to efficiently remove even the smallest viruses, i.e., up to 99.9980–99.9995% efficiency, at industrially relevant flow rates, i.e., 60–500 L m^−2^ h^−1^, and low fouling, i.e., *V*_max_ > 10^3^–10^4^ L m^−2^. The filter paper presented in this work shows great promise for the development of robust, affordable, and sustainable water purification systems.

## 1 Introduction

Urbanization and population growth lead to increased and inhomogeneous consumption of water resources. The problem of drinking water is very complex and multifaceted, and, therefore, it is relevant for both high‐ and low‐income countries. This is why access to safe drinking water and sanitation is among the UN's Sustainable Development Goals, i.e., Agenda 2030.^1^ In low‐income countries, when a centralized water supply is not available or the quantity is limited, poor households have to rely on water obtained from rivers, rain, or shallow wells.^2^ The lack of safe water is a part of a vicious cycle, where poverty and poor healthcare feed each other. Water safety is also an important issue in high‐income countries with centralized water supply since water‐borne outbreaks continue to occur^3^, ^4^, ^5^, ^6^, ^7^, ^8^, ^9^, ^10^ and many more incidents are expected, especially among susceptible population groups, including increasing elderly population above the age of 65, children below the age of 5, chronically ill patients, recipients of immunosuppressive therapies, and pregnant women.^11^ Globalization of commerce, expansion of food supply sources from regions with unimproved irrigation, people migration from areas with poor hygiene, and natural sporadic mutations contribute to the spread of water‐borne diseases.^11^

There are about 140 known water‐borne pathogens, including viruses, bacteria, and protozoa. Viruses are the smallest and probably the most difficult to deal with from them all. They have the greatest infectivity among all known water‐borne pathogens and high resistivity to disinfection.^11^ They are further excreted with feces in relatively large quantities (>10^11^ g^−1^) and generally exhibit longest survival potential in the environment. It is estimated that known viral pathogens account for nearly 8% of water‐borne infection outbreaks. It should however be noted that water‐borne infections of undetermined etiology, which account for almost half of all documented cases, are probably due to viruses for which currently there are no suitable laboratory host cultures.^11^ According to the World Health Organization (WHO), moderately to severely infectious water‐borne viral pathogens are numerous and include adenovirus, astrovirus, echovirus, hepatitis viruses, rotavirus, caliciviruses (including norovirus), enteroviruses (including coxsackieviruses and polioviruses), polyomaviruses, cytomegalovirus, papillomaviruses, influenza viruses, and coronaviruses.^12^, ^13^, ^14^ Massive virus outbreaks due to poor water quality can occur in any country, including countries with centralized water supply and sustained vigilance.^3^, ^4^, ^5^, ^6^, ^7^, ^8^, ^9^, ^10^ Only in the USA, there are estimated 4 million to 33 million cases of acute gastrointestinal illnesses each year due to drinking water.^15^, ^16^ Virus clearance is more difficult than that of bacteria, and, therefore, bacteriologically safe water may not be necessarily virally safe.

Traditionally, the main source for water‐borne pathogens has been considered to be surface water, since all surface waters contain water‐borne pathogens, with a substantial number of zoonotic infections. Animal farms and sewage discharges in close proximity are well‐known sources of contamination for surface water.^11^ Recently, it is recognized that another potential source of virus contamination, previously considered safe and therefore unmonitored, is groundwater, which is utilized directly by small communities and private homeowners. Several surveys in the USA documented evidence of viruses in ground water,^17^, ^18^, ^19^ which is often associated with proximity of septic systems to water sources due to landfill leaks, improper wastewater disposal, and septic tank contamination.^11^ It is estimated that about half of the water‐borne disease outbreaks in the USA is due to untreated groundwater.^19^ Also in the European Union (EU), where there are ≈85 000 decentralized small supplies providing drinking water to ≈65 million citizens, almost 40% of them are not properly monitored or do not deliver drinking water complying with microbiological quality standards.^20^

In water treatment, virus inactivation, aimed at reducing the infectivity of viruses, can be achieved both by physical (e.g., UV‐C and γ‐irradiation and boiling) and chemical means (e.g., oxidants and coagulants).^21^, ^22^ Chemical disinfection, e.g., with chlorine, is the most common way of disinfection. However, higher doses are normally required to destroy virus as compared to bacteria,^21^, ^22^, ^23^ and chlorination may also lead to toxic by‐products (e.g., trihalomethanes and haloacetic acids) as well as residual infectivity depending on the water quality.^24^ An increased reliance on UV light for water disinfection has raised the concerns for viral resistance, especially related to adenovirus infection,^25^, ^26^ which is especially problematic for immunocompromised population, e.g., in cancer and bone marrow transplant patients.^11^ Chemical coagulation using metal ions, e.g., alum (Al_2_(SO_4_)_3_), salts of iron, lime (Ca(OH)_2_), and various polyelectrolytes is the second most common method of inactivating viruses from water after disinfection.^21^, ^22^, ^27^, ^28^, ^29^ However, research suggests that flocculation does not remove all viruses exhaustively, showing varying results between log_10_ virus titer reduction of 1 and 2.86.^30^

Size‐exclusion filtration is a very robust and highly efficient method of clearing viruses, which does not require using chemicals, heat, or radiation. Most common filters, including ceramic‐ and biosand‐based filters, used in water treatment, while effective against bacteria (typically >6log_10_ reduction) feature low virus removal capacity (typically, <1log_10_ reduction).^31^ Therefore, to be able to completely remove viruses from water, only advanced filters can be used featuring pore sizes of around 10–20 nm. In this respect, affordable and efficient ultrafilters, which are preferably produced from sustainable raw materials, i.e., nonplastic, are highly demanded.

In the context of above discussion, cellulose‐based filters for water treatment would be useful due to the abundance, sustainability, and cost efficiency of this raw material.^32^ Traditional general‐purpose filter paper has too large pores to remove bacteria or viruses from water.^33^ Various adsorptive‐type virus retentive cellulose‐based depth filters have been developed.^34^, ^35^ However, because viruses possess highly variable isoelectric points, ranging between 1.9 and 8.4,^36^ adsorptive‐type virus retentive depth filters show limited robustness and their function can be interfered by other competing charged species.^37^ On the other hand, the size‐exclusion virus removal filters based on cuprammonium‐regenerated cellulose, e.g., Planova filters by Asahi Kasei, used for purification of protein‐based drugs and which are manufactured by phase inversion processing,^34^ remain unaffordable for water treatment applications due to their high price.

Virus removal filters should combine high flow rates, low fouling, and high virus removal capacity, which explains the high price of such filters. Controlling the pore‐size distribution of paper filter in the region suitable for virus removal is not easy. This is because during drying nanocellulose irreversibly agglomerates into a compact, impermeable film due to a process known as “cellulose hornification.”^38^ Alternatively, solvent exchange, critical point drying, or freeze‐drying can be used to produce cellulose aerogels but these are expensive processes, too, for industrial‐scale production.^38^

The advances in nanocellulose science allow today for cost‐efficient production of this material.^39^ Recently, a highly cost‐efficient virus removal filter paper, aka mille‐feuille filter paper, was described for applications in biotechnology.^40^, ^41^ It is produced by traditional paper‐making processing, which contrasts starkly from the membrane technology relying on phase inversion. This nonwoven filter paper, made from 100% cellulose nanofibers, features a stratified internal architecture, consisting of numerous cellulose nanofiber sheets—hence, its name is “mille‐feuille” (or thousand leaves) filter paper. The separation efficiency of the mille‐feuille filter was verified for surrogate nanoparticles, e.g., gold nanoparticles or fluorophore‐labeled latex nanobeads,^41^, ^42^, ^43^ and real viruses, e.g., influenza virus, i.e., swine influenza A virus (100 nm);^41^ retrovirus, i.e., murine leukemia virus (100 nm);^44^ and parvovirus, i.e., minute virus of mice (20 nm).^40^ In this article, the properties of the mille‐feuille filter paper are evaluated for water treatment applications for the first time. Furthermore, this work discusses the effect of process parameters such as filtration pressure and virus load on clearance efficiency.

## 2 Results and Discussion

### 2.1 Pore‐Size Characterization of Nanocellulose Filter Papers

**Figure 1:**
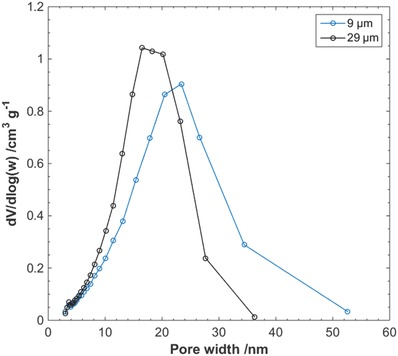
BJH N_2_ gas desorption pore‐size distribution of nanocellulose filter papers.

The pore‐size distribution of the nanocellulose filter papers was evaluated both in the dry state, using nitrogen gas sorption, and in the wet state, using differential scanning calorimetry (DSC) cryoporometry (CP‐DSC). The resulting pore‐size distributions from nitrogen sorption measurements of the nanocellulose filter papers of thicknesses 9 and 29 µm are presented in **Figure** **1**. A shift in the pore‐size distribution toward pores of a greater width was observed for the thinner filter papers, consistent with previous observations.^40^, ^42^

The results from CP‐DSC of the nanocellulose filter papers of thicknesses 9 and 29 µm are summed up in **Table** **1**, where peak temperature for melting of water confined in pores, temperature depression, and calculated peak pore mode radius for the nanocellulose filter papers is presented. No statistically significant difference in the peak pore mode radius, *r*_p_, between the two filter thicknesses was found from the CP‐DSC measurements. Representative heat flow curves from CP‐DSC measurements of the nanocellulose filter papers can be found in Figure S1 in the Supporting Information.

**Table 1:** CP‐DSC analysis of nanocellulose filter papers

Filter paper thickness [µm]	*T*_pk_ a) [°C]	*ΔT*_on‐pk_ b) [°C]	Peak pore mode radius, *r*_p_ [nm]
9	−1.06 ± 0.08	−1.67 ± 0.08	13.5 ± 0.7
29	−1.03 ± 0.08	−1.64 ± 0.08	13.7 ± 0.6

^a)^ *T*_pk:_ Peak temperature for melting of water confined in pores

^b)^ Δ*T*_on‐pk_: Temperature depression.

### 2.2 Simulated Waste Water Filtration

**Figure 2:**
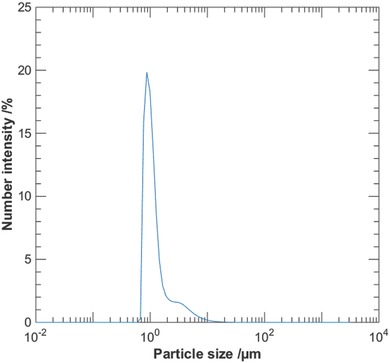
Particle‐size distribution from dynamic light scattering analysis of SWW. The distribution has a peak value of *d*_particle_ = 0.87 µm and no particles were found at *d*_particle_ ≤ 0.59 µm and *d*_particle_ ≥ 31.1 µm.

The nanocellulose filter papers were challenged with simulated waste water (SWW) dispersions containing two different amounts of total suspended solids (TSS), i.e., 0.251 and 2.51 mg L^−1^. The source of the TSS in the SWW was diatomite and formazin was the turbidity contributing additive. Dynamic light scattering (DLS) analysis of the particle diameter, *d*_particle_, in the SWW revealed a particle size distribution with a peak value of 0.87 µm for the suspended solids, as presented in **Figure** **2**. No particles at cutoff values *d*_particle_ ≤ 0.59 µm and *d*_particle_ ≥ 31.1 µm were found.

**Figure 3:**
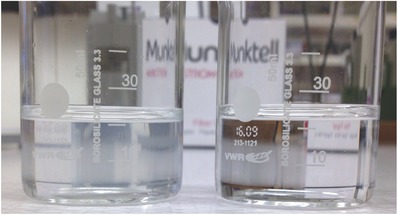
Feed (left) and permeate (right) in filtrations of SWW through nanocellulose filter papers. The total solid content of the feed dispersion was 0.251 mg L^−1^.

Compared to the feed dispersion, a visible decrease in the turbidity of the permeate was noticed in the filtrations of SWW for both TSS contents. In **Figure** **3** representative pictures of feed and permeate are presented.

**Figure 4:**
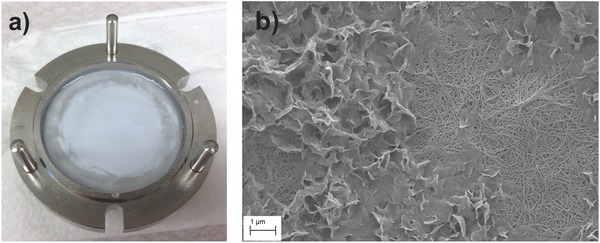
a) Postfiltration image of a nanocellulose filter paper challenged with SWW with a TSS content of 2.51 mg L^−1^. b) SEM image of a nanocellulose filter paper postfiltration of SWW. Deposited solids from the SWW are visible on top of the filter structure. The image was taken at 21 × 10^3^ times magnification.

As seen in **Figure** **4**a, a clear accumulation of solids on the filter surface was observed after filtration of SWW. From the scanning electron microscopy (SEM) image presented in Figure 4b, the solid deposit on the filter surface does not appear to be clogging the porous structure of the filter, and exposed areas of the filter are visible through the deposited solids.

The measured transmittance at 500 nm of feed and collected permeates is presented in **Tables** **2** and **3**, where *T*_permeate_ and *T*_feed_ are the measured transmittances for permeate and feed, corrected for water background.

**Table 2:** Measured transmittance for SWW filtrations through nanocellulose filter papers. TSS content was 0.251 mg L^−1^. *T* = 100% corresponds to the transmittance of pure water

Filter paper thickness [µm]	Overhead pressure [bar]	*T*_feed_^500 nm^ [%]	*T*_permeate_^500 nm^ [%]
9	1	94.7 ± 0.4	100 ± 0
9	3	94.9 ± 0.3	100 ± 0
29	1	94.2 ± 0.6	100 ± 0
29	3	94.5 ± 0.1	100 ± 0

**Table 3:** Measured transmittance for SWW filtrations through nanocellulose filter papers. TSS content was 2.51 mg L^−1^

Filter paper thickness [µm]	Overhead pressure [bar]	*T*_feed_^500 nm^ [%]	*T*_permeate_^500 nm^ [%]
9	1	46.9 ± 0.6	100 ± 0.0
9	3	46.9 ± 0.6	100 ± 0.0
29	1	45.7 ± 1.7	100 ± 0.1
29	3	45.7 ± 1.7	100 ± 0.1

The transmittance measurements suggest that all solids were removed from the SWW when filtered through the nanocellulose filter paper, in accordance with what was observed in Figure 4a and in SEM postfiltration imaging in Figure 4b. Considering the particle distribution in the SWW, where no particles where found at *d*_particle_ ≤ 0.59 µm, no particles should be small enough to pass the nanometer‐sized pores of the nanocellulose filter paper.

**Figure 5:**
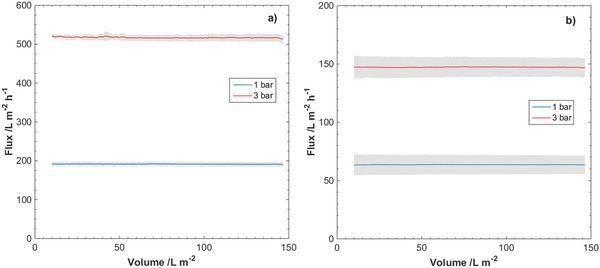
Observed fluxes at overhead pressures 1 and 3 bar for filtrations of SWW through nanocellulose filter papers of thicknesses a) 9 µm and b) 29 µm. TSS content in SWW was 0.251 mg L^−1^. Gray area indicates standard deviation of the measured flux for three separate filtrations.

**Figure 6:**
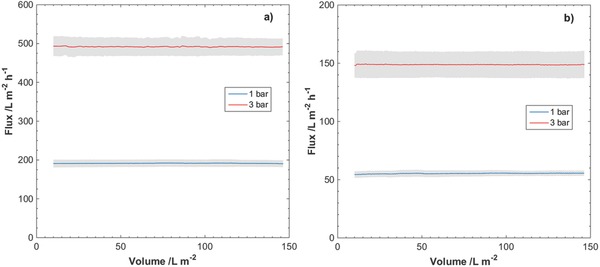
Observed fluxes at overhead pressures 1 and 3 bar for filtrations of SWW through nanocellulose filter papers of thicknesses a) 9 µm and b) 29 µm. TSS content in SWW was 2.51 mg L^−1^. Gray area indicates standard deviation of the measured flux for three separate filtrations.

The observed fluxes during filtrations of SWW through the nanocellulose filter papers are presented in **Figures** **5** and **6**. The manufacturer recommends the TSS content of 0.251 mg L^−1^ for validation studies. No or little decline in flux was observed during filtration at the reported pressures and amounts of TSS. The flux seemingly remained unaffected by the amount of TSS in the feed dispersion, i.e., 0.251 and 2.51 mg L^−1^. The average flux values for 9 µm filter papers at 1 and 3 bar were 200 and 500 L m^−2^ h^−1^, respectively. The average flux values for 29 µm filter papers at 1 and 3 bar were 65 and 150 L m^−2^ h^−1^, respectively.

The fouling behavior during filtration of the SWW was further analyzed and quantified through the so‐called *V*_max_ analysis.^45^ *V*_max_ was calculated from the slope of the linear fit associated with each flux data curve and is available in Figures S2 and S3 as well as Tables S1 and S2 in the Supporting Information.

As seen from both the flux data presented in Figures 5 and 6 as well as the result of the *V*_max_ analysis, the flux remained stable during filtration of SWW through the nanocellulose filter paper. This would indicate that no significant clogging of the filter occurs when challenged with the SWW. When a value for *V*_max_ was possible to achieve, i.e., a positive value for 1/*V*_max_, the *V*_max_ analysis suggests that volumes in the order of 10^4^ L m^−2^ can be filtered before complete clogging of the nanocellulose filter paper occurs.

### 2.3 Filtration of Latex Nanoparticles in SWW

**Figure 7:**
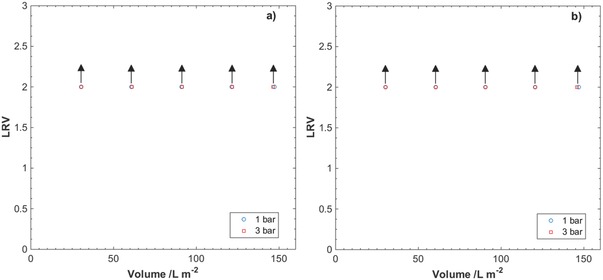
Logarithm reduction values (LRV) for filtration of 30 nm latex particles in SWW through nanocellulose filter papers of thicknesses a) 9 µm and b) 29 µm. Arrows indicate that the obtained values are below the limit of detection (LOD) in fluorescence spectroscopy of the 30 nm latex particles. The results are the average of two measurements.

The nanocellulose filter papers were further challenged with SWW dispersions containing latex particles with a diameter of 30 nm. The TSS content in the dispersions was 0.251 mg L^−1^ and the concentration of 30 nm latex particles was in the order of 10^11^ mL^−1^. The resulting removal efficiency of the 30 nm latex particles is presented in **Figure** **7** for pressures 1 and 3 bar, and for filter thicknesses 9 and 29 µm. The limit of detection (LOD) in fluorescence spectroscopy of the 30 nm latex particles corresponds to a logarithmic reduction value (LRV) of 2.0, and no latex particles where detected in permeates at the reported pressures for neither of the two filter thicknesses.

**Figure 8:**
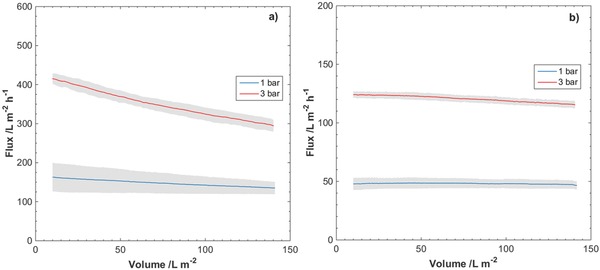
Observed fluxes at overhead pressures 1 and 3 bar for filtrations of 30 nm latex particles in SWW through nanocellulose filter papers of thicknesses a) 9 µm and b) 29 µm. Gray area indicates standard deviation of the measured flux for two separate filtrations.

The observed fluxes during filtrations of 30 nm latex particles in SWW are presented in **Figure** **8**. In contrast to the filtrations performed with only SWW, a decline in the flux can be observed for the thinner filter papers at both pressures and for the thicker filter papers at 3 bar.

A *V*_max_ analysis was performed for the flux data presented in Figure 8 and the result is shown in Figure S4, available in the Supporting Information. Slope of the linear fit and the calculated value for *V*_max_ for the different filter thicknesses and pressures are summarized in **Table** **4**.

**Table 4:** Results from *V*_max_ analysis for filtration of 30 nm latex particles in SWW through nanocellulose filter papers

Filter paper thickness [µm]	Filtration overhead pressure [bar]	Slope of linear fit [1/*V*_max_]	*V*_max_ a) [L m^−2^]
9	1	1.3 (±0.6) × 10^−3^	9.7 (±4.6) × 10^2^
9	3	2.5 (±0.1) × 10^−3^	4.0 (±0.2) × 10^2^
29	1	1.4 × 10−4 b)	7.4 × 103 b)
29	3	5.7 (±0.3) × 10^−4^	1.8 (±0.1) × 10^3^

^a)^ Values for *V*_max_ were obtained at a particle concentration of 10^11^ mL^−1^

^b)^ The second filtration exhibited a negative slope in the *V*_max_ analysis.

From the results of the *V*_max_ analysis presented in Table 4, a tendency of fouling behavior occurring at a faster rate can be seen for the thinner filters, as compared to the thicker filters. Another interesting result from the *V*_max_ analysis is that fouling tends to occur at a faster rate when the operational flux is higher. In particular, the calculated *V*_max_ at 3 bar for the 9 µm filter papers is lower compared to at 1 bar. The same observation can be made with regard to the 29 µm filter papers, where the calculated *V*_max_ is lower at 3 bar compared to at 1 bar. The calculated *V*_max_ values were significantly lower for the 9 µm filter papers compared to the 29 µm filter papers.

### 2.4 Filtration of ΦX174 Bacteriophage in SWW

**Figure 9:**
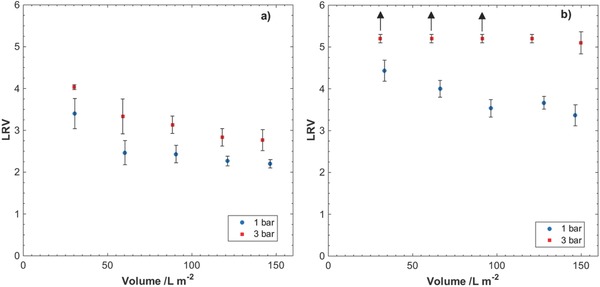
Logarithm reduction values (LRV) for filtration of ΦX174 bacteriophages in SWW through nanocellulose filter papers of thicknesses a) 9 µm and b) 29 µm at overhead pressures 1 and 3 bar. Arrows indicate that obtained values are below the limit of detection (LOD) in the plaque‐forming unit (PFU) assay, and no phage particles were detected in large volume plating. The results are the average of three measurements.

The nanocellulose filter papers were also challenged with SWW dispersions spiked with the 28 nm large ΦX174 bacteriophage, used as a surrogate model for worst‐case small‐sized mammalian viruses.^46^, ^47^, ^48^ TSS content of the dispersions was 0.251 mg L^−1^ and the concentration of ΦX174 bacteriophages was in the order of 10^6^ mL^−1^. The resulting LRVs for the filtrations are presented in **Figure** **9**, and the virus removal efficiency is summed up in **Table** **5**.

**Table 5:** Virus removal efficiencies for filtration of ΦX174 bacteriophages in SWW through nanocellulose filter papers

Filter paper thickness [µm]	Filtration overhead pressure [bar]	Load, ≈3 L m^−2^ [%]	Load, ≈58 L m^−2^ [%]	Load, ≈93 L m^−2^ [%]	Load, ≈133 L m^−2^ [%]	Load, ≈148 L m^−2^ [%]
9	1	99.9700	99.5000	99.6800	99.3700	99.2100
		99.9800	99.8400	99.7500	99.6000	99.5000
		99.9000	99.5000	99.3700	99.3700	99.3700
9	3	99.9900	99.9000	99.8700	99.7500	99.6800
		99.9900	99.9800	99.9500	99.9000	99.9000
		99.9900	99.9400	99.9400	99.8700	99.8400
29	1	99.9940	99.9900	99.9700	99.9800	99.9200
		99.9980	99.9940	99.9800	99.9800	99.9700
		99.9960	99.9800	99.9500	99.9700	99.9600
29	3	99.9995	99.9995	99.9995	99.9995	99.9995
		99.9994	99.9994	99.9994	99.9994	99.9994
		99.9992	99.9992	99.9992	99.9992	99.9980

As seen from the results presented in Figure 9 and Table 5, thinner filter papers exhibit lower virus removal efficiency compared to the thicker filters. In general, better removal efficiency for both filter thicknesses is achieved at the higher overhead pressure, i.e., 3 bar. It is further seen from Figure 9 that the efficiency of removal is progressively decreasing with increased load, especially for 9 µm filter papers. Nonetheless, even for the thinner filter at 1 bar, the removal efficiency never falls below 99.2% for the largest load volumes.

Under the experimental conditions presented in this work, two cases should be scrutinized in more detail, i.e., 9 µm filter paper at 1 bar and 29 µm filter paper at 3 bar. Both filters show comparable pore‐size modes and flow rates at respective overhead pressure, i.e., 200 L m^−2^ h^−1^ for 9 µm filter paper at 1 bar and 150 L m^−2^ h^−1^ for 29 µm filter paper at 3 bar. However, the performance of these two filter papers with respect to virus removal efficiency and fouling is very different. In particular, the 29 µm filter paper at 3 bar exhibits steady LRV ≥ 5 and *V*_max_ ≈ (1.8 ± 0.1) × 10^3^ L m^−2^, measured with spiked 30 nm latex nanoparticles in SWW, whereas for 9 µm filter paper at 1 bar the observed virus LRV gradually decreases from 3.5 to 2.2 and *V*_max_ is around (9.7 ± 4.6) × 10^2^ L m^−2^, under similar experimental conditions. The latter difference could potentially be due to compaction of the 29 µm filter paper at 3 bar as discussed earlier.^40^ It should be noted that the 9 µm filter exhibits higher variability of *V*_max_ values, which is likely to be due to larger spread of initial flux values and extrapolation.

The presented data using SWW show that nanocellulose filter paper has the capacity to efficiently remove even the smallest viruses at industrially relevant flow rates and low fouling. Thus, it is expected that the filter can act as a total microorganism removal filter, removing not only viruses but also all other larger microbes. Compared to other available advanced water treatment membranes, the mille‐feuille filter is attractive for point‐of‐use applications as an affordable, single‐use filter paper, similar to coffee filters used in household for brewing. Because traditional water treatment membranes are used over an extended period of time, i.e., 5–15 years after installation, they need to be regularly backflashed and treated with chemical disinfectants (hypochlorite) in order to avoid biofilm formation on the surface of the membrane. The biofilm formation is a major issue for the membranes, as it is deteriorating the key processing parameters over time, including flux and permeate quality. In contrast, an affordable single‐use filter paper with excessive microorganism removal capacity and high flux eliminates the risk of biofilm formation and thereby the need for regular cleaning and disinfection. Therefore, we suggest that the mille‐feuille filter paper is an interesting point‐of‐use, sustainable alternative to address the global challenges related to drinking water purification.

## 3 Conclusion

In this work, the filtration of SWW matrix spiked with surrogate latex nanobeads and ΦΧ174 bacteriophages as model for worst‐case small‐sized mammalian virus is shown using the nanocellulose‐based filter paper for drinking water purification applications. The presented data using SWW matrix show for the first time that a filter paper made from 100% nanocellulose is capable of efficiently removing even the smallest viruses, i.e., up to 99.9980–99.9995% efficiency, at industrially relevant flow rates, i.e., 60–500 L m^−2^ h^−1^, and low fouling, i.e., *V*_max_ > 10^3^–10^4^ L m^−2^. The filter paper presented here shows great promise for development of robust, affordable, and sustainable water purification systems.

## 4 Experimental Section

*Materials: Cladophora* algae cellulose was provided by FMC BioPolymer (batch G3828‐112). SWW matrix was obtained from Sigma (MMW001‐250ML, lot number LRAB5412). Fluorescent latex beads (30 nm; carboxylate‐modified polystyrene; L5155), calcium chloride dihydrate (C5080), magnesium chloride hexahydrate (M2670), and sodium chloride (S5886) were purchased from Sigma‐Aldrich.

*Biological Materials: Escherichia coli* (Migula) Castellani and Chalmers (*E. coli*, 13 706) bacteria strain and ΦΧ174 bacteriophage (13 706‐B1) were purchased from the American Type Culture Collection (ATCC). Agar (214 530) and yeast extract (212 750) were purchased from BD. Tryptone broth powder (T‐broth) (J870) was purchased from Amresco.

*Preparation of Nanocellulose Filter Paper*: A dispersion of *Cladophora* cellulose (0.1 wt%) was prepared. The dispersion was then run twice in succession through 200 and 100 µm sized chambers at 1800 bar, using an LM20 Microfluidizer. Filter papers of two different thicknesses, i.e., 9 and 29 µm, were then prepared by adjusting the solids content of dispersion. The resulting nanocellulose dispersion was then drained over a nylon filter membrane (Durapore, 0.65 µm DVPP, Merck Millipore) fitted in a funnel using vacuum. The resulting wet cellulose mass was then dried at 80 °C using a hot press (Carver, USA).

*Nitrogen Gas Sorption*: Pore‐size distribution evaluation of the nanocellulose filter papers with the Barret–Joyner–Halenda (BJH) method^49^ was performed based on the desorption branch of the nitrogen sorption isotherm. This was done using an ASAP 2020 (Micrometrics, USA) instrument. The filter sample was degassed at 90 °C in vacuum for 4 h and then analyzed by nitrogen sorption, carried out at 77 K. The performance of the instrument was validated using Micrometrics Silica‐Alumina SSA 210 m^2^ g^−1^ (lot number: A‐501‐49) standard prior to analysis. The deviation between the pore‐size mode of the calibration data from the nominal standard values was 0 nm.

*Cryoporometry DSC*: Pore‐size distribution evaluation of the nanocellulose filters was performed using cryoporometry DSC, using a Mettler Toledo DSC 3 (Switzerland). The filter samples were soaked in water for 1 h prior to DSC analysis to ensure substantial wetting of the filter structure. DSC analysis was performed by cooling the samples to −25 °C at a rate of 15 K min^−1^ followed by heating of the samples to 4 °C at a rate of 0.7 K min^−1^. Five separate measurements were performed for each filter thickness. The peak value for the melting of bulk water was determined from repeated measurements on deionized water. Deionized water (1, 2, 4, 6, and 10 µL) was analyzed in five separate measurements per volume, and the mean peak value for the water was determined to be 0.61 °C.

Cryoporometry is a method for determining the pore size in porous materials from the liquid–solid transformation of a probing medium. With the use of DSC, the pore radius can be related to the temperature where freezing or melting of a probing liquid in the pores occur.^50^ If melting of the probing liquid is considered, transition from solid to liquid phase confined in pores will occur at a temperature *T* below the melting temperature *T*_m_ of the bulk liquid. This temperature depression, Δ*T*, is related to the pore radius *r*_p_ as shown in Equation (1).^51^

(1) $$\Delta T\;\;\approx \;\;\frac{2T_{0}\gamma_{\mathrm{sl}}}{\rho_{l}\Delta H_{f}}\frac{\cos\theta }{r_{p}}$$

*T*_0_ is the melting temperature of the liquid, γ_sl_ is the surface tension between the solid and liquid phases, ρ_l_ is the density of the liquid, *ΔH*_f_ is the heat of fusion for the liquid, *ϴ* is the contact angle between the solid and liquid phases. Landry^51^ performed cryoporometry measurements on controlled‐pore glass samples with water as the probing liquid and found the following empirical expression for the pore radius as a function of the temperature depression during melting:

(2) $$r_{p}\;\left(\mathrm{nm}\right)\;\;=\;\;-\frac{19.082}{\Delta T+0.1207}\;\;+\;\;1.12$$

The peak pore mode radius was determined from the DSC, using Equation (2). The difference between the peak maximum for melting of pore confined solid phase and the peak value for melting of bulk water (0.38 °C), Δ*T*_on‐pk_, was used as the temperature depression.

*Dynamic Light Scattering*: Particle‐size distribution of the suspended solids in the SWW was analyzed through DLS using a Malvern Mastersizer 3000. Refractive index of the dispersant was set to that of water, i.e., 1.33, and the refractive index of the particles was set to that of diatomite, i.e., 1.43.^52^ Particle geometry was set as nonspherical, and Mie scattering^53^ was used as the scattering model. The manufacturer recommended TSS content was used (0.251 mg L^−1^).

*Filter Thickness Evaluation*: The thickness of the manufactured filter papers was evaluated using a Mitutoyo Absolute digital caliper (ID‐C150XB) with a precision of 1 µm. The thickness was measured for the two different filter thicknesses on five different filters at five different positions on each filter.

*Filtration of SWW*: Filtration of SWW was carried out using an Advantec KST‐47 filter holder. The feed volume and filtering surface area were only limited by the choice of available filter holder without other limitations. The nanocellulose filter papers were fitted in the cell using a Munktell General Purpose Filter Paper as a mechanical support. The filters were wetted with deionized water prior to filtration.

Feed dispersions of SWW were prepared by dilution (2 and 20 mL, respectively) of SWW‐simulated matrix with deionized water to a final volume (200 mL). Final TSS contents were achieved (0.251 and 2.51 mg L^−1^). Filtrations were carried out at two different overhead pressures, i.e., 1 or 3 bar. The permeate solution was collected during the experiment, and the real flux was monitored using a scale (Mettler Toledo, MS1602TS) registering the change in weight of the collected permeate solution over time. The absorption of the collected permeate was analyzed at 500 nm using a Shimadzu UV‐1650 PC spectrophotometer.

*Scanning Electron Microscopy*: Postfiltration imaging of the nanocellulose filter papers was performed using a scanning electron microscope (LEO1550, Zeiss, Germany). The filters were sputtered prior to SEM analysis with Au/Pt at 2 kV, 25 mA for 35 s to avoid charging of the material. SEM pictures were retrieved at acceleration voltages of 1.00 and 1.50 kV.

*Filtration of Latex Nanoparticles in SWW*: Filtration of 30 nm fluorescent latex particles in SWW was carried out using an Advantec KST‐47 filter holder. The nanocellulose filter papers were fitted with a Munktell General Purpose Filter Paper as a mechanical support in the cell, and the filters were then prewetted with deionized water. Feed dispersions were prepared by dilution (2 mL) of SWW‐simulated matrix and 30 nm fluorescent latex particles (80 µL) with deionized water (total volume of 200 mL). Filtrations were carried out at overhead pressures 1 and 3 bar. The permeate solution was collected in fractions (40 mL) each, and the real flux was monitored using a scale (Mettler Toledo, MS1602TS) registering the change in weight of the collected permeate solution over time. The fluorescence of the collected permeate fractions was measured between 450 and 580 nm, with an excitation wavelength of 264 nm, using a TECAN M200 spectrophotometer. The area under the curve (AUC) was calculated for the fluorescence peak, after subtraction of background emission from water, by integration of the measured wavelengths. The particle removal rate was described by the LRV and was calculated using Equation (3).

(3) $$\mathrm{LRV}\;=\;\;\log_{10}\;\frac{{\mathrm{AUC}}_{\mathrm{feed}}}{{\mathrm{AUC}}_{\mathrm{permeate}}}$$

AUC_feed_ is the area under the curve for the feed solution and AUC_permeate_ is the area under the curve for the permeate solution.

*Filtration of ΦX174 Bacteriophages in SWW*: The ΦX174 bacteriophage was propagated by inoculation of *E. coli* host bacteria at exponential growth phase in Luria–Bertani medium (1% tryptone‐*t* broth, 0.5% yeast extract, 1% NaCl, 1 × 10^−3^ m CaCl_2_·2H_2_O, and 1 × 10^−3^ m MgCl_2_·6H_2_O in deionized water) for 5 h at 35 °C with an agitation (120 rpm). Bacteriophage was harvested by centrifugation at 5000 ×*g* for 10 min, and the suspension was collected and stored at 4 °C prior to use.

The ΦX174 bacteriophage titer was determined by plaque‐forming units (PFU) assay and expressed as PFU mL^−1^. Briefly, to perform an assay, tenfold dilutions of a bacteriophage stock were prepared and inoculated into *E. coli* host on the agar plates. Each dilution was plated in duplicate to enhance the accuracy, and PFU mL^−1^ was calculated using Equation (4).

(4) $$\log_{10}\left(\frac{\mathrm{PFU}}{\mathrm{mL}}\right)\;\;=\;\;\log_{10}\left(\frac{\mathrm{number}\;\mathrm{of}\;\mathrm{plaques}}{0.1\;\;\times \;\;\mathrm{dilution}\;\mathrm{factor}}\right)$$

where 0.1 is the volume (mL) of the added virus.

To supplant the air from the inner pores of the nanocellulose‐based filter, prefiltration with deionized water was performed. The filter was fixed in an Advantec KST‐47 filter holder, 30 mL of deionized water was added, and the filtration was driven by the overhead air pressure of 1 bar. Prefiltration was stopped after 20 mL of the water was filtered to avoid the ingress of the air into the filter.

SWW was spiked with ΦX174 bacteriophage stock dispersion to obtain the titer of ≈10^6^ PFU mL^−1^. Feed solution (200 mL) was filtered through the nanocellulose‐based filter at the overhead air pressure of 3 bar in a dead‐end setup. Permeate samples were collected (40 mL fractions) and stored at 4 °C before PFU assay. Bacteriophage removal capacity was expressed by LRV

(5) $$\mathrm{LRV}\;=\;\;\log_{10}\;{\left(\frac{\mathrm{PFU}}{\mathrm{mL}}\right)}_{\mathrm{feed}}\;-\;\log_{10}{\left(\frac{\mathrm{PFU}}{\mathrm{mL}}\right)}_{\mathrm{permeate}}$$

The limit of detection (≤0.7 PFU mL^−1^) of PFU assay current experimental design refers to ≤5 bacteriophages mL^−1^, corresponding to a single detectable plaque in one of the plates for nondiluted duplicate samples. To detect any possibly omitted bacteriophage in the permeate fractions, the filtrates were concentrated by centrifuging at 4500 × *g* in 100 kDa cutoff protein concentrator tubes (Thermo, 88 533) to the final dead stop volume (≈50 µL). Concentrates were inoculated into *E. coli* host culture at exponential growth phase in Luria–Bertani medium for 5 h at 35 °C with an agitation (120 rpm). Bacteriophage presence was determined by decreased OD_600_ values compare to control samples.

## Conflict of Interest

The corresponding author is the inventor behind IP related to the virus removal filter paper.

## Supporting information

Supplementary
