# Supplementary material for: High‐Performance Virus Removal Filter Paper for Drinking Water Purification
Source: Glob Chall. 2018 Jul 11;2(7):1800031. doi: 10.1002/gch2.201800031 (PMC6607295; doi:10.1002/gch2.201800031)
Supplement: Supplementary file 1 — Supplementary [file GCH2-2-1800031-s001.pdf]

Copyright WILEY-VCH Verlag GmbH & Co. KGaA, 69469 Weinheim, Germany, 2018.

Supporting Information

for *Global Challenges,* DOI: 10.1002/gch2.201800031

High-Performance Virus Removal Filter Paper for Drinking Water Purification

*Olof Gustafsson, Levon Manukyan, and Albert Mihranyan**

Supporting Information

High Performance Virus Removal Filter Paper for Drinking Water Purification

Olof Gustafsson, Levon Manukyan, Albert Mihranyan

CP-DSC. Typical heat flow curves from CP-DSC analysis of the nanocellulose filter papers are presented in Figure S1.

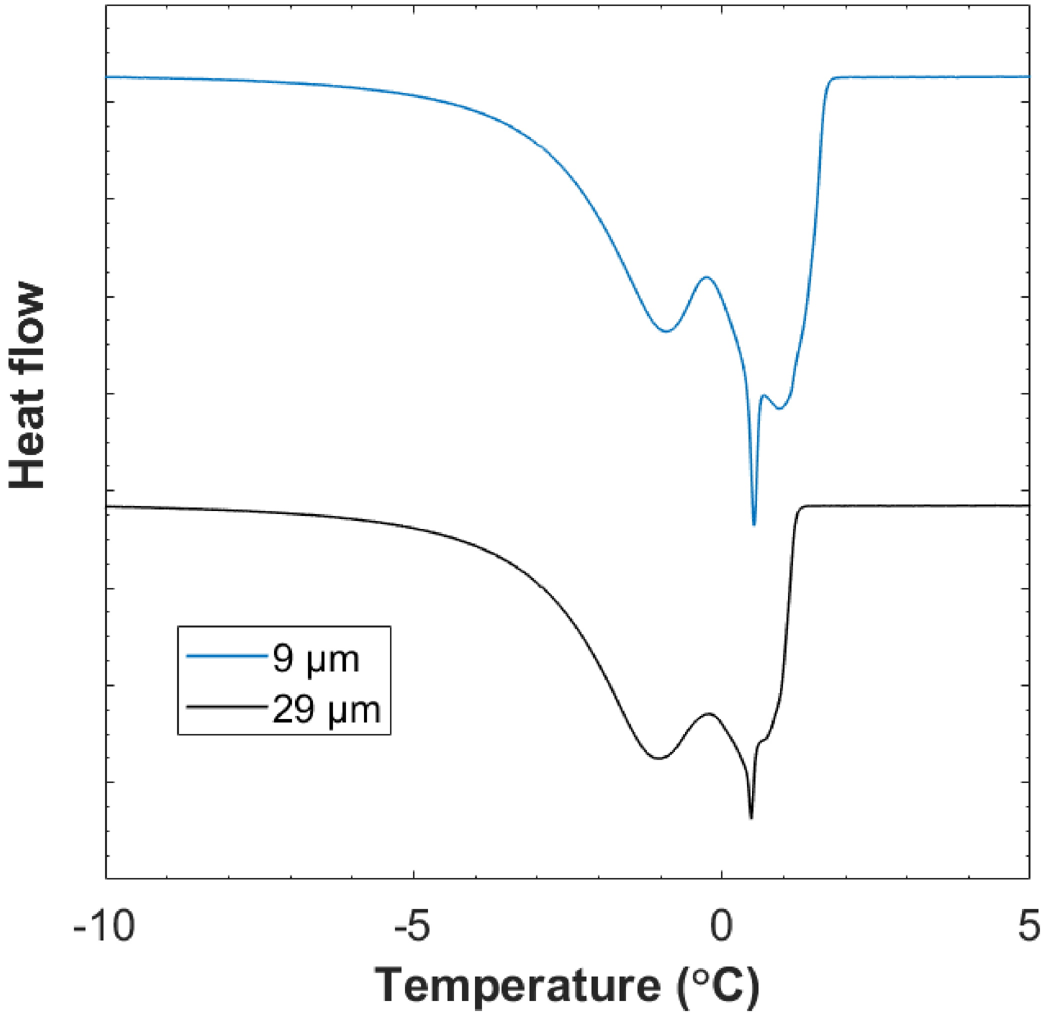

Figure S1. Typical heat flow curves as a function of temperature for nanocellulose filter papers of thicknesses 9 µm and 29 µm acquired from DSC with water as liquid and a heating rate of 0.7 K/min.

Two peaks are distinguishable in the figure for both thicknesses; i.e. one peak for melting of bulk water around 0.5 °C and another peak for melting of water confined in pores around -1 °C.

SWW Filtration. The V_{max} analysis is based on the linearized form of the flux decay model, with pore constriction as the fouling mechanism, expressed in Equation S1.

$$\frac{1}{Q} = \frac{1}{Q_0} + \left(\frac{1}{V_{max}}\right) t \qquad \text{(S1)}$$

Q is the measured flux over time t, and Q_o is the initial flux. V_{max} is the maximum throughput volume before complete clogging of the filter structure occurs. V_{max} is given from the slope when $1/Q$ is plotted against t, and is a result of extrapolation of the experimental flux data.

In Figure S3 and S4 the resulting V_{max} analysis of the flux data presented in Figure 5 and 6 is shown. For negative slopes, V_{max} was reported as not available (N/A), due to a resulting negative value for V_{max}.

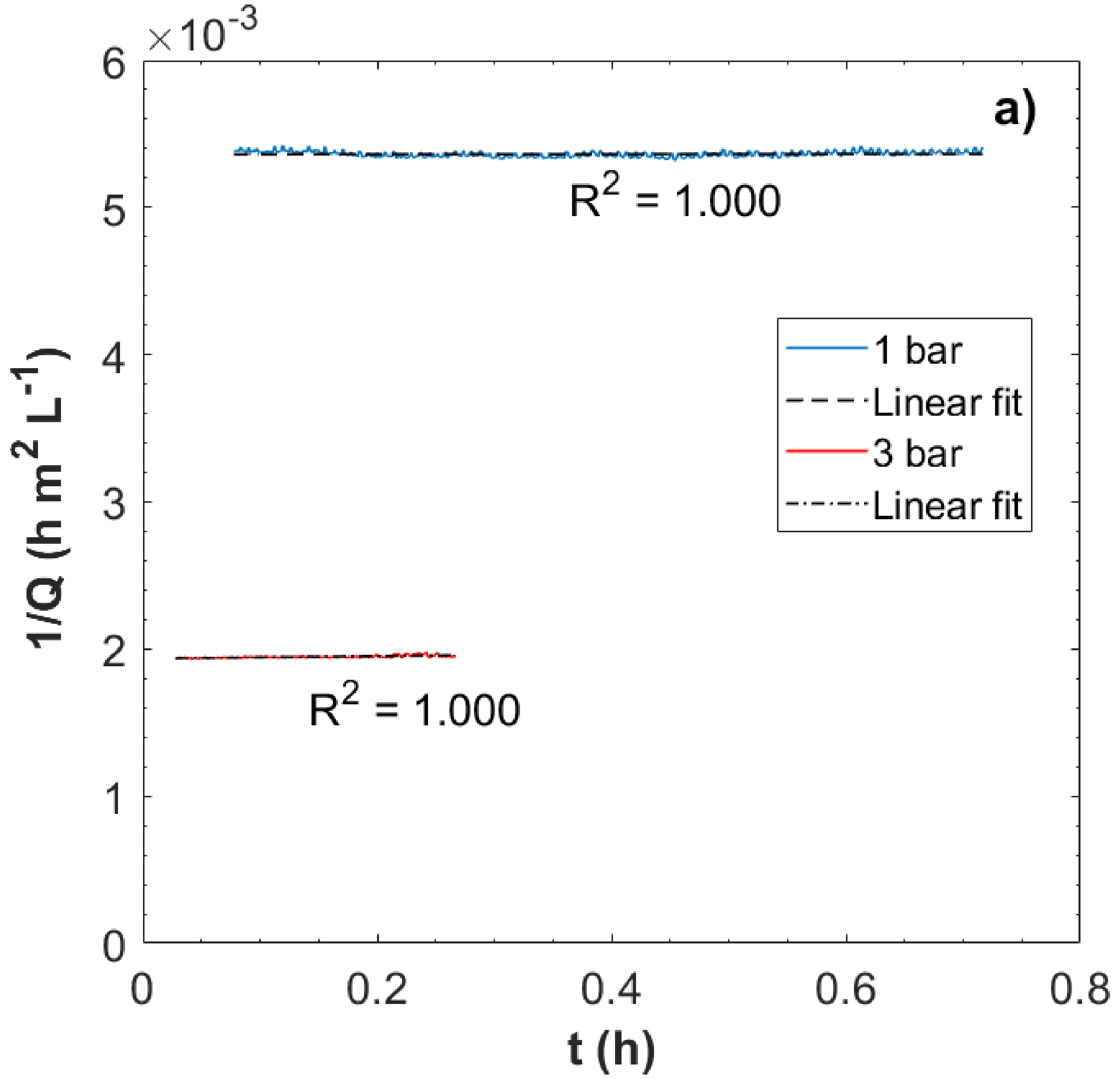
×10^-3
a)
R^2 = 1.000
1 bar
Linear fit
3 bar
Linear fit
R^2 = 1.000
1/Q (h m^2 L^-1)
t (h)
0
1
2
3
4
5
6
0
0.2
0.4
0.6
0.8

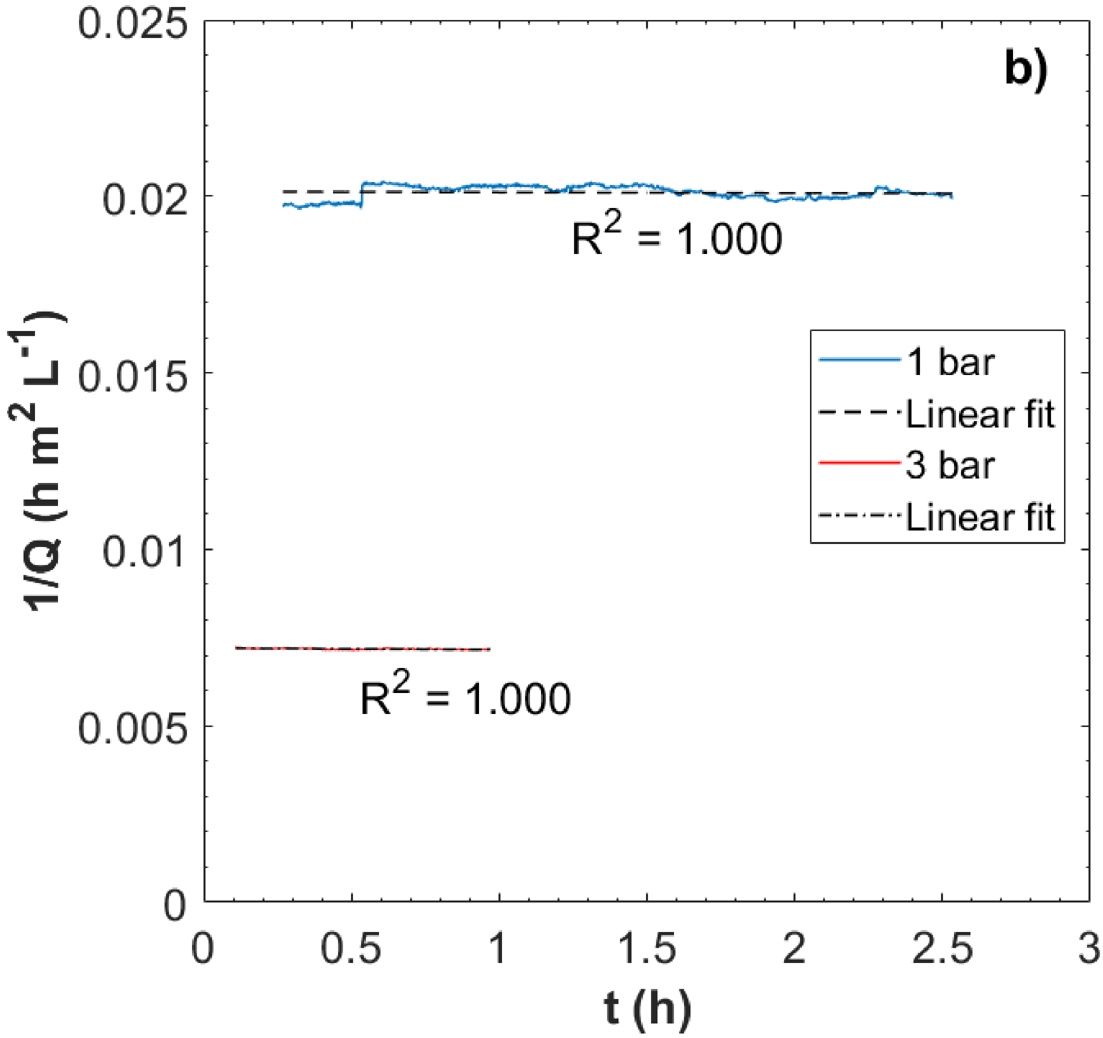

Figure S2. Typical curves from V_{max} analysis for filtration of SWW through nanocellulose filter papers of thicknesses a) 9 µm and b) 29 µm. Solid curves indicate observed values of the inverted flux at overhead pressures 1 bar and 3 bar. The dashed curves are linear regression fits for analysis of V_{max} according to Equation S1. The TSS content in the SWW was 0.251 mg/L.

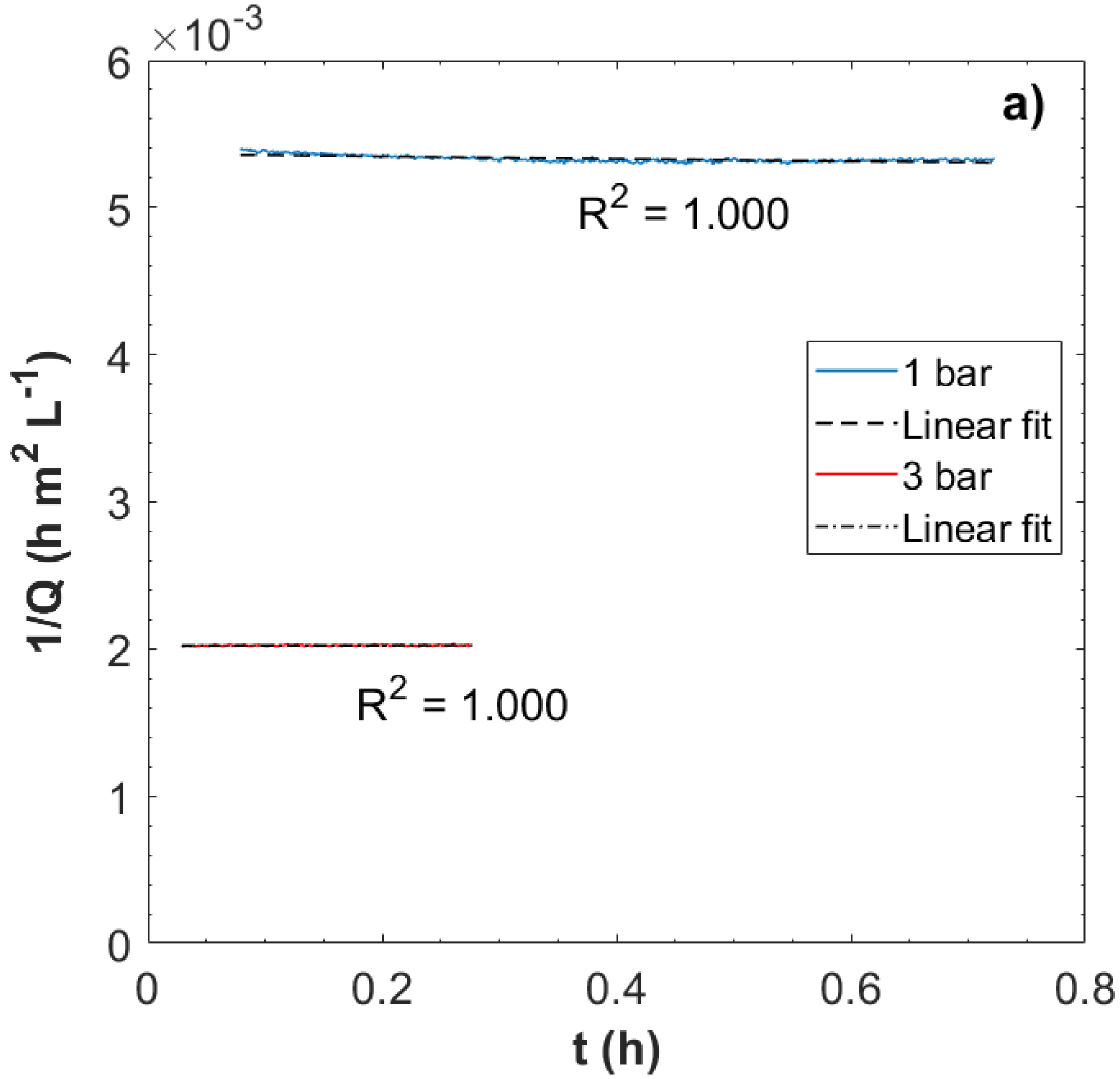

×10^-3
a)
R^2 = 1.000
R^2 = 1.000
1 bar
Linear fit
3 bar
Linear fit
1/Q (h m^2 L^-1)
t (h)
0
1
2
3
4
5
6
0
0.2
0.4
0.6
0.8

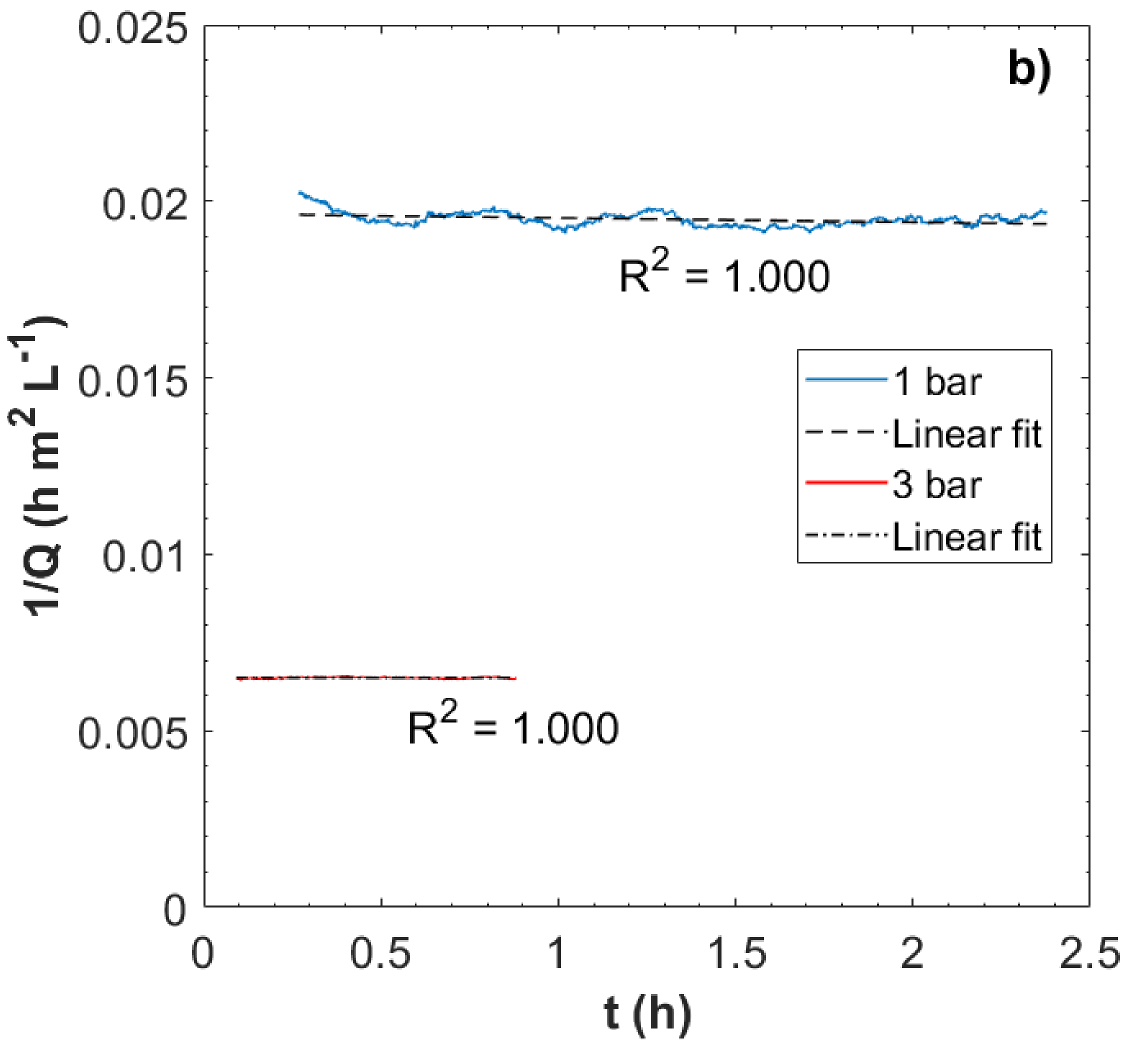

Figure S3. Typical curves from V_{max} analysis for filtration of SWW through nanocellulose filter papers of thicknesses a) 9 µm and b) 29 µm. Solid curves indicate observed values of the inverted flux at overhead pressures 1 bar and 3 bar. The dashed curves are linear regression fits for analysis of V_{max} according to Equation S1. The TSS content in the SWW was 2.51 mg/L.

Table S1. Results from V_{max} analysis for filtration of SWW through nanocellulose filter papers. The TSS content in the SWW was 0.251 mg/L.

Filter paper thickness [µm]	Filtration overhead pressure [bar]	Slope of linear fit [1/V_{max}]	V_{max} [L m^{-2}]
9	1	$1.0 \cdot 10^{-4}$	$1.0 \cdot 10^{4}$
	1	$4.7 \cdot 10^{-6}$	$2.1 \cdot 10^{5}$
	1	$-2.7 \cdot 10^{-6}$	N/A
9	3	$8.1 \cdot 10^{-5}$	$1.2 \cdot 10^{4}$
	3	$4.3 \cdot 10^{-5}$	$2.3 \cdot 10^{4}$
	3	$-7.8 \cdot 10^{-5}$	N/A

29	1	$-1.9 \cdot 10^{-5}$	N/A
	1	$9.3 \cdot 10^{-5}$	$1.1 \cdot 10^{4}$
	1	$-4.7 \cdot 10^{-4}$	N/A
29	3	$-7.7 \cdot 10^{-5}$	N/A
	3	$-2.0 \cdot 10^{-4}$	N/A
	3	$-4.2 \cdot 10^{-5}$	N/A

Table S2. Results from V_{max} analysis for filtration of SWW through nanocellulose filter papers. The TSS content in the SWW was 2.51 mg/L.

Filter paper thickness [µm]	Filtration overhead pressure [bar]	Slope of linear fit [$1/V_{max}$]	V_{max} [L m^{-2}]
9	1	$-5.0 \cdot 10^{-6}$	N/A
	1	$-8.3 \cdot 10^{-5}$	N/A
	1	$4.4 \cdot 10^{-5}$	$2.3 \cdot 10^{4}$
9	3	$-3.5 \cdot 10^{-5}$	N/A
	3	$5.9 \cdot 10^{-5}$	$1.7 \cdot 10^{4}$
	3	$1.1 \cdot 10^{-5}$	$9.3 \cdot 10^{4}$
29	1	$-1.2 \cdot 10^{-4}$	N/A
	1	$-2.9 \cdot 10^{-4}$	N/A
	1	$-4.6 \cdot 10^{-4}$	N/A
29	3	$1.9 \cdot 10^{-5}$	$5.1 \cdot 10^{4}$
	3	$2.3 \cdot 10^{-5}$	$4.3 \cdot 10^{4}$
	3	$-4.0 \cdot 10^{-6}$	N/A

Filtration of Latex Nanoparticles in SWW. Typical curves from the V_{max} analysis from filtrations of 30 nm latex particles in SWW is presented in Figure S4. The linear fittings showed good correlations to experimental data in the V_{max} analysis for both filter thicknesses and pressures, as seen from the R^2-values in Figure S4. This would indicate that pore constriction is the fouling mechanism during filtration of the latex particles in the filter paper.

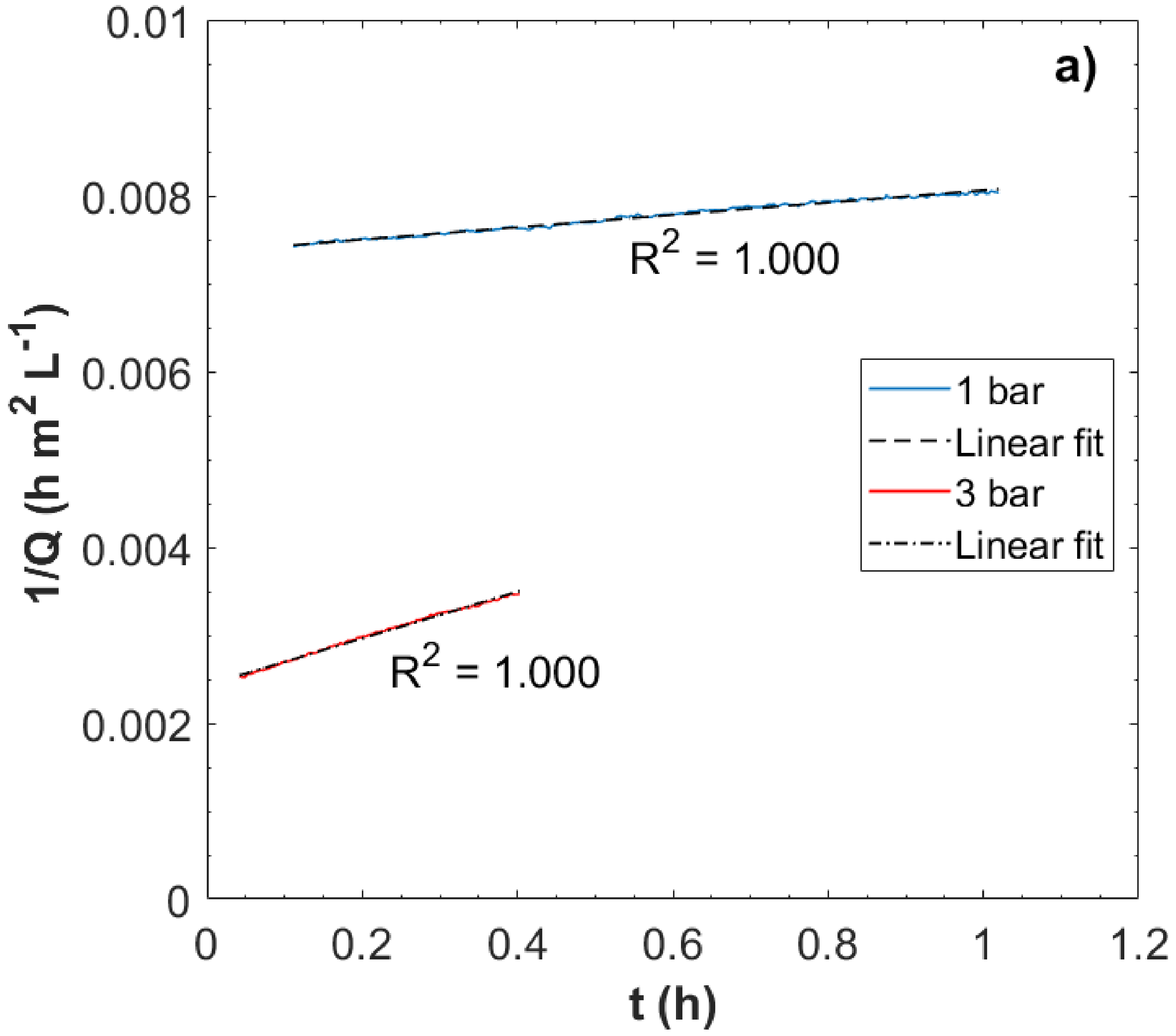
a)
R^2 = 1.000
R^2 = 1.000
1 bar
Linear fit
3 bar
Linear fit
1/Q (h m^2 L^{-1})
t (h)
0.01
0.008
0.006
0.004
0.002
0
0
0.2
0.4
0.6
0.8
1
1.2

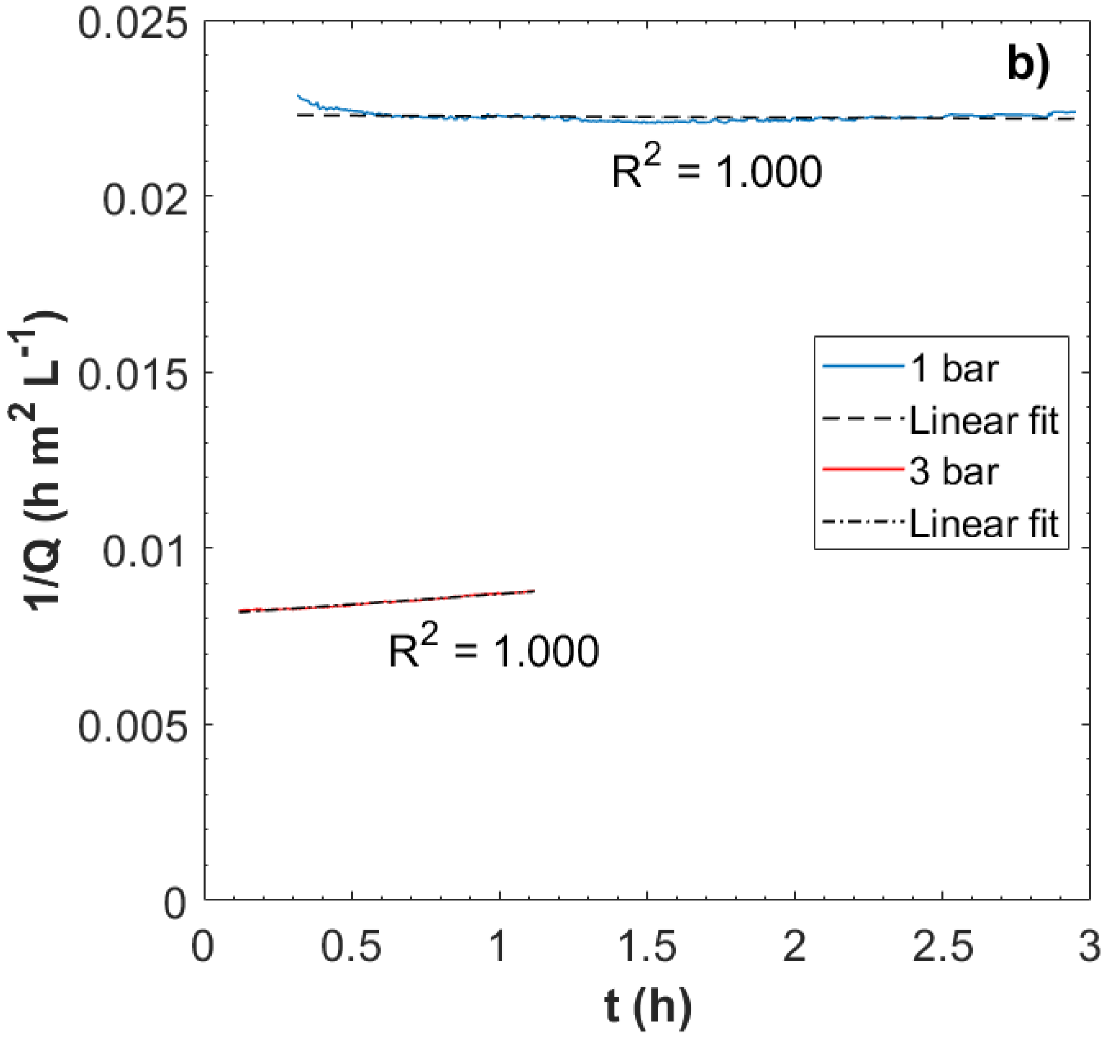

Figure S4. Typical curves from V_{max} analysis for filtration of 30 nm latex particles in SWW through nanocellulose filter papers of thicknesses a) 9 µm and b) 29 µm. Solid curves indicate observed values of the inverted flux at overhead pressures 1 bar and 3 bar. The dashed curves are linear regression fits for analysis of V_{max} according to Equation S1.
